# Lentil protein and trehalose conjugates: Structural interactions and mechanisms for improving multi‐level structure and functional characteristics

**DOI:** 10.1111/1750-3841.17465

**Published:** 2024-10-22

**Authors:** Mohammad Alrosan, Sofyan Maghaydah, Ali Al‐Qaisi, Ali Madi Almajwal, Muhammad H. Alu'datt, Farah R. Al Qudsi, Thuan‐Chew Tan, Ammar A. Razzak Mahmood

**Affiliations:** ^1^ Department of Food Science and Nutrition, Faculty of Agricultur Jerash University Jerash Jordan; ^2^ Applied Science Research Center Applied Science Private University, Al‐Arab St. 21, 11931 Amman Jordan; ^3^ QU Health College of Health Sciences Qatar University Doha Qatar; ^4^ Department of Human Nutrition and Dietetics, College of Health Sciences Abu Dhabi University, Zayed City Abu Dhabi UAE; ^5^ Department of Nutrition and Food Technology, Faculty of Agriculture Jordan University of Science and Technology Irbid Jordan; ^6^ Department of Agricultural Biotechnology, Faculty of Agricultural Sciences and Technology Palestine Technical University—Kadoorie (PTUK) Tulkarm Palestine; ^7^ Department of Community Health Sciences, College of Applied Medical Sciences King Saud University Riyadh Saudi Arabia; ^8^ Department of Food Science & Nutrition, College of Life Sciences Kuwait University Safat Kuwait; ^9^ Department of Food Science University of Guelph Guelph Ontario Canada; ^10^ Food Technology Division, School of Industrial Technology Universiti Sains Malaysia USM Penang Malaysia; ^11^ Department of Pharmaceutical Chemistry College of Pharmacy‐University of Baghdad Baghdad Iraq

**Keywords:** digestibility, lentil proteins, protein structure, solubility, trehalose

## Abstract

**Abstract:**

This study aimed to improve lentil proteins’ (LPs) functionality and nutritional value, specifically addressing their lower water solubility and digestibility. A unique combination of LP‐disaccharide interactions was employed. Spectroscopic technologies, which include fluorescence spectra, ultraviolet spectra, and Fourier‐transform infrared, investigated the structure of LPs at various concentrations of trehalose. The results indicate that the LP structures and conformation were considerably modified (*p *< 0.05) following trehalose conjugation. The surface charge and hydrophobicity of the trehalose‐conjugated LPs (T‐LPs) were significantly altered (*p *< 0.05), from −22.7 to −31.4 and 753 and 543 a.u., respectively. Furthermore, the digestibility and solubility of T‐LPs increased from 75% to 81.8% and 60% to 66%, respectively. In conclusion, this study showed that combining LPs and trehalose conjugation could improve the quality of conjugates LPs, which could expand their use in manufacturing as the acceptance of plant‐based diets increases.

**Practical Application:**

Currently, lentil proteins (LPs) are used in plant‐based protein powders and supplements. Though less popular than soy or pea proteins, LPs are valued for their high protein content and good amino acid profile. LPs are utilized in meat alternatives and high‐protein snack products. The application of these products is mainly due to their nutritional benefits rather than functional properties due to their poor water solubility. Increasing the water solubility of LPs could significantly expand their application in various food industries, making LPs a more competitive and functional plant‐based protein source. Trehalose‐conjugated LPs with better water solubility allow LPs to be used in other food products, such as plant‐based protein beverages. Better solubility would enhance the clarity and smoothness of these products, making them more appealing to consumers.

## 1 INTRODUCTION

Protein digestibility is critical in determining protein quality because it breaks down the protein into amino acids for absorption and utilization (Orlien et al., 2023). Plant‐based proteins have lower digestibility: lentil proteins (LPs, 76.42%, Alrosan et al., 2021), quinoa proteins (78.54%, Alrosan et al., 2022a), and chickpea proteins (63.62%, Liu et al., 2023), compared to animal‐based proteins: whey (88.48%, Alrosan et al., 2022a) and casein (83.7%, Almeida et al., 2015). Several factors affect plant‐based protein digestibility, including the amino acid profile, fiber content, anti‐nutritional agents, extraction process, and physical form (Jarpa‐Parra et al., 2017). LPs are predominantly globulins and contain certain anti‐nutritional factors, such as phytic acid and enzyme inhibitors (Jarpa‐Parra et al., 2014, 2017).

Protein solubility in water strongly connects to their functional properties, including gelation, emulsification, foaming, viscosity, and protein interaction (Grossmann & McClements, 2023; Jayakody et al., 2023). In addition, the water solubility of proteins is a crucial factor in affecting their protein digestibility (Alrosan et al., 2024) by affecting enzymatic access, surface area for enzymatic action, gastric emptying rate, and nutrient absorption in the gastrointestinal tract. Liu et al. (2023) and Alrosan et al. (2024) demonstrated that protein conformation and multi‐structure of proteins can affect digestibility and water solubility. Protein conjugates formed through interactions with polysaccharides (Jiang et al., 2022) or disaccharides (Alrosan et al., 2024) can significantly improve protein functionality. Trehalose is a disaccharide that occurs in nature and comprises two molecules of glucose linked by chemical bonds. Trehalose is utilized in various capacities in technological advancement and medicinal products. Trehalose has hydroxyl groups on its glucose molecules, allowing them to be involved in hydrogen bonds. The hydroxyl groups in a molecule can establish hydrogen bonds between other particles, including protein structures. Hydrogen bonding between trehalose and proteins helps stabilize the protein's tertiary and quaternary structures. It was reported by Wang et al. (2023) that combining trehalose, sucrose, or maltodextrin with egg yolk powder in a 5% ratio resulted in modifications to the protein properties, leading to increased resistance of the protein structure and improved water solubility of the conjugates. The solubility of the conjugates rose from 15% to 56% after their conjugation with trehalose at 5%. Trehalose can interact with ions in its surroundings through electrostatic interactions. The electrostatic attraction between trehalose and ions can influence the overall stability of the complex and may affect processes such as ion sequestration or ion‐mediated aggregation.

The widespread consumption of proteins produced from soybean, whey, and wheat, although advantageous in various capacities (Alrosan et al., 2022a; Zhao et al., 2020), have a significant disadvantage, that is, they are allergens. Soybean, whey, and wheat proteins are among the common allergens that can trigger reactions ranging from mild intolerance to severe allergic reactions, that is, anaphylaxis (Alrosan et al., 2022a; Zhao et al., 2020). This limits their use in foods intended for people with allergies or sensitivities to these ingredients. On the other hand, the dietary restrictions for whey proteins are unsuitable for vegans and individuals with lactose intolerance (Alcorta et al., 2021). Similarly, wheat proteins are not a good option for celiac disease or gluten sensitivities (Cabanillas, 2020). Soy proteins can be problematic for avoiding soy due to hormonal concerns or soy allergies (Swallah et al., 2023). Moreover, the nutritional limitations of soybean, whey, and wheat are rich in certain nutrients, which might not provide as balanced a profile of amino acids as needed for some diets (Alcorta et al., 2021; Almeida et al., 2015; Alrosan et al., 2022a; Cabanillas, 2020; Zhao et al., 2020). For instance, whey proteins are rich in essential amino acids. Still, they are relatively low in other nutrients compared to plant‐based proteins that offer fiber and a variety of vitamins and minerals (Alrosan et al., 2022b).

Legumes are extremely suitable for the synthesis of protein isolates because of their high protein content, low cost, and general acceptability (Alrosan et al., 2022b; Jarpa‐Parra et al., 2017; Jarpa‐Parra et al., 2014). Recently, LPs have been increasingly utilized as functional ingredients in food products due to their exceptional nutritional and sensory properties. LPs are particularly effective in enhancing the nutritional quality of foods by adding essential amino acids and health‐promoting polypeptides (Martínez‐Villaluenga et al., 2024). These components are crucial for overall health, supporting muscle repair and growth, and promoting optimal bodily functions. This study aims to investigate an innovative approach incorporating LPs with trehalose based on the pH‐shifting technique. Several studies reported that pH‐shifting techniques significantly enhance the characteristics of the protein structure of protein and reflect on the water solubility of protein (Alrosan et al., 2022b; Jiang et al., 2022). The purpose is to determine the potential benefits of this combination for preparing a soluble plant protein composite with high nutritional values. By analyzing the effects of LPs and trehalose on various biophysical and biochemical parameters, this study aims to provide valuable insights into improving the multi‐structure of LPs and preparing high nutritional values and functionality of composite compounds.

## 2 MATERIALS AND METHODS

### 2.1 Materials

The lentil grains (*Lens culinaris*) were bought from a local market in Jordan. LP was prepared through alkaline (pH 9.5) extraction, followed by isoelectric precipitation at a pH of 4.2. This step succeeded by neutralization, lyophilization, and milling to get fine lyophilized LP powder (Jarpa‐Parra et al., 2014) with protein content around 71.11% ± 1.43 based on the AOAC Kjeldahl Method 930.29 (Association of Official Analytical Chemists [AOAC], 2012). All supplementary chemicals used in this investigation were high purity and obtained from Sigma‐Aldrich. The obtained trehalose had a molecular weight of 378.33 g/mol.

#### 2.1.1 Preparation of trehalose solutions

Trehalose solutions were produced at 0%, 1%, 2%, 3%, and 5% (w/w) in a phosphate‐buffered solution. These solutions were indicated with the following designations: 0T, 1T, 2T, 3T, and 5T, respectively. A magnetic mixer (OHAUS, Guardian 5000) was used to mix the trehalose solutions for 2 h at room temperature (21°C).

#### 2.1.2 Trehalose conjugation

A magnetic stirrer dissolved 1 g of LPs in 100 mL of trehalose solution to create the LP‐trehalose conjugate suspensions (w/v, 1%). The LP‐trehalose suspensions were stirred for 4 h at pH 7.0. To inhibit the growth of microorganisms, sodium azide (0.002%, w/v) was added to each suspension and left overnight at 4°C. The following day, the mixtures were stirred for 1 h at pH 12 (pH adjusted with 1 M NaOH) before being changed to a neutral state (pH 7.0) using 1 M HCl. Subsequently, the LP‐trehalose conjugates were allowed to rest overnight at 4°C. Supernatants were obtained by centrifuging (CN Meditech, CNME060222) the mixture for 10 min at 7000 × *g*. The following characterization was carried out using the dried supernatants from the freeze‐dryer.

### 2.2 Analyses of LP‐trehalose conjugates

#### 2.2.1 Protein digestibility

The protein digestibility of the trehalose‐conjugated LPs (T‐LPs) was measured using the method reported by Alrosan et al. (2024) with a few modifications. The samples (250 mg) were combined with pepsin (385 units/mg) at a 1.5 mg/mL concentration and then dispersed in a solution containing 15 mL of 0.1 M HCl. The solution was subsequently placed in a water bath (Memmert, WB22) and incubated at 37°C for 3 h. A 0.005 M solution of sodium azide, approximately 1 mL, was added to the protein mixture to inhibit the growth of bacteria. Following incubation, the samples were treated with 7.5 mL of 0.5 M NaOH, and pancreatins (10 mg) of 0.2 M phosphate buffer (pH 8.0) were added to the mixture and incubated overnight at 37°C. After that, 1 mL of trichloroacetic acid (10%) was added to the mixture, followed by centrifugation at 10,000 × *g* for 20 min using the CNME060222 centrifuge. The nitrogen content of the samples before and after centrifugation was determined using the AOAC Kjeldahl Method 930.29 (AOAC, 2012). Protein digestibility of the samples was calculated using the following equation:

(1) $$\mathrm{Digestibility}\;\;\left(\%\right)=\frac{N_{S}-N_{B}}{N_{T}}\times 100\%$$

where *N*_S_, *N*_T_, and N_B_ represent the nitrogen content of the supernatant, sample before centrifugation, and blank, respectively.

#### 2.2.2 Water solubility

The water solubility of the LPs in varied concentrations of trehalose solutions was measured by measuring nitrogen content in samples (Alrosan et al., 2024 1‐anilinonaphthalene‐8‐sulfonic acid). Samples (200 mg) were mixed with 188 mL of distilled water. The pH of the mixtures was adjusted to 7.0 using 0.1 M HCl. Following the pH modification, the mixtures were stirred continuously at 1000 rpm with the Guardian 5000 magnetic stirrer for 1 h. Right before the end of the stirring, the mixtures were adjusted to 1% (w/v) using distilled water. The mixtures were centrifuged at 10,000 × *g* for 20 min using the CNME060222 centrifuge. The Kjeldahl method (AOAC Method 930.29) was used to determine the nitrogen content of the mixtures before and after centrifugation (supernatant) and blank (AOAC, 2012):

(2) $$\mathrm{Water}\;\mathrm{solubility}\;\;\left(\%\right)=\frac{N_{S\;}-\;N_{B}}{N_{T}}\times 100\%$$

where *N*_S_, *N*_T_, and N_B_ represent the nitrogen content of the supernatant, sample before centrifugation, and blank, respectively.

#### 2.2.3 Fourier‐transform infrared (FT‐IR) spectroscopy

A Fourier‐transform infrared (FT‐IR) spectrometer (Thermo Scientific, Smart iTR) was used to identify the secondary protein components of the T‐LPs. The region around 1600–1699 cm^−1^ in the FT‐IR spectrum associated with the amide I band was analyzed on the basis of the method described by Alrosan et al. (2021). The data values were obtained by calculating the mean of three measurements.

#### 2.2.4 Intrinsic fluorescence

The tertiary protein structures of the T‐LPs were determined by investigating the intrinsic fluorescence of tryptophane using a fluorescence spectrophotometer (Agilent, Cary Eclipse). At a wavelength range emission (300–450 nm) and excitation (280 nm), the was used to perform the intrinsic fluorescence (Wang et al., 2023).

#### 2.2.5 UV‐spectrometry

The ultraviolet spectra (ranging between 190 and 350 nm) of the T‐LP were obtained using a UV‐vis spectrophotometer (Shimadzu, UV‐1650 PC) based on the method described by Liu et al. (2023). To produce combinations with a concentration of 0.01% (w/v) at pH 7.0 at room temperature (21°C), samples were dissolved into distilled water and mixed simultaneously.

#### 2.2.6 Non‐covalent interactions

The Cary Eclipse fluorescence spectrophotometer's intrinsic fluorescence was used to determine the non‐covalent interactions of the T‐LPs. For the measurement of the hydrophobic interactions, electrostatic interactions, and hydrogen bonding of the samples, T‐LPs were mixed with 10 mM sodium dodecyl sulfate, NaCl, and thiourea, respectively (Wang et al., 2019), before measuring the intrinsic fluorescence using the Cary Eclipse fluorescence spectrophotometer.

#### 2.2.7 Surface hydrophobicity

The 1‐ anilinonaphthalene‐8 sulfonic acid (ANS) was employed as a fluorescence instrument in the solution to assess the T‐LP and LPs’ surface hydrophobicity. The technique employed depends on the one provided by Johnston et al. (2015). The proteins identified in the T‐LPs were diluted using a phosphate‐buffered solution, and the concentration range was from 0.01% to 0.1%. Each diluted dispersion of the T‐LPs (4 mL) was added to 20 µL of an 8 mM solution of ANS. The resulting emission and excitation were then recorded between 470 and 390 nm, respectively, with a slit width of around 1 nm, using the Cary Eclipse fluorescence spectrophotometer to analyze the fluorescence properties of the T‐LPs. Based on the slope of the plotted graphs (relative fluorescence intensity against protein content), the T‐LPs’ surface hydrophobicity was measured and determined.

#### 2.2.8 Surface charge

A zetasizer (Malvern Panalytical, Mastersizer 2000) was used to ascertain the surface charge of the T‐LPs following the methodology outlined by Wang et al. (2019). A concentration of 1 mg was achieved by preparing the samples in distilled water. It has been determined that the protein samples and the distilled water each have a refractive index of 1.450 and 1.330, respectively.

#### 2.2.9 Particle size

The particle size of the T‐LPs was assessed using the Mastersizer 2000 zetasizer. The solutions were prepared by diluting the sample in distilled water to a concentration of 1 mg/mL. Subsequently, 1 mL of the solutions was injected into the device for analysis. Each value of the data was obtained as the average of three measurements.

#### 2.2.10 Differential scanning calorimetry (DSC)

The thermal stability of LPs and LP conjugates was examined using a differential scanning calorimetry (DSC) (TA Instruments, DSC Q200). The samples were prepared by weighing them before being placed in a chamber purged with nitrogen to ensure dryness. For each measurement, two aluminum crucible pots were used: One that was empty, serving as a reference, and another that contained 5 mg of powdered LPs and LP conjugates. The device was set up with high‐purity nitrogen as the carrier gas at a 100 mL/min flow rate. The samples were first balanced at 25°C for 3 min, through a cooling process from 25 to −70°C at a 20°C/min rate. The samples were then heated to 150°C at a similar rate. A DSC program was used to analyze the temperature of denaturation (*T*_d_) derived from the thermal spectrum analysis.

### 2.3 Statistical analysis

SPSS version 23.0 was employed to conduct the statistical analysis. One‐way analysis of variance was performed to analyze the data. Duncan's multiple range test was used to compare the means for multiple comparisons.

## 3 RESULTS AND DISCUSSION

### 3.1 Digestibility

**FIGURE 1:**
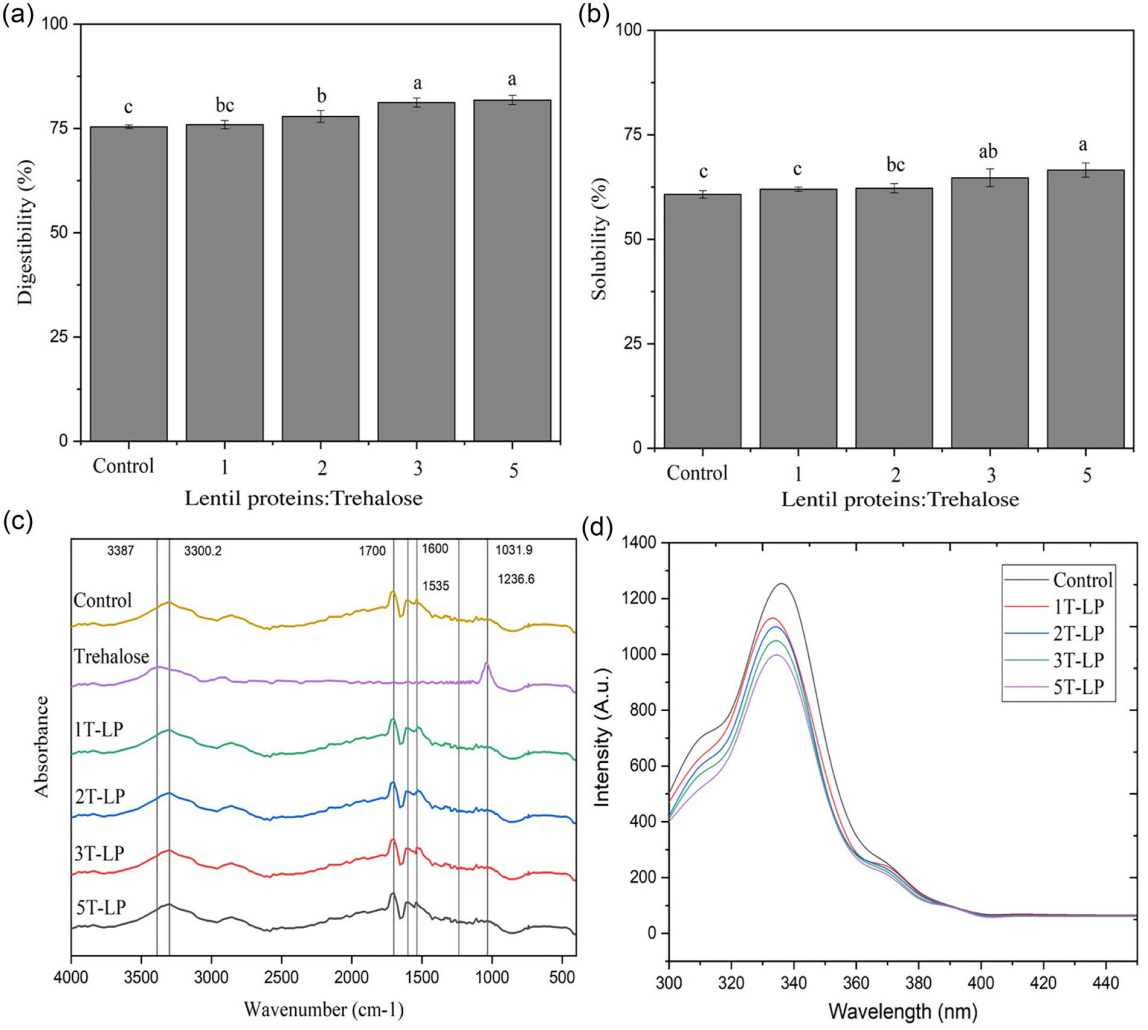
The trehalose‐conjugated lentil proteins (LPs) were evaluated for alterations in the (a) protein digestibility, (b) water solubility, (c) Fourier‐transform infrared (FT‐IR) absorbance, and (d) fluorescence intensity. LPs that have been conjugated with trehalose at 1, 2, 3, and 5% (w/w) are represented by the 1T‐LP, 2T‐LP, 3T‐LP, and 5T‐LP, respectively, whereas the control (0T‐LP) indicates that no trehalose conjugation with LPs is present.

Protein digestibility directly affects proteins’ absorption and utilization in food applications (Alrosan et al., 2022a). A higher protein digestibility indicates that the protein is more easily digested and provides a greater availability of amino acids for absorption and utilization by the body. Conversely, a lower digestibility value suggests that the protein is less digestible and may provide fewer amino acids. The digestibility of LPs demonstrated a similar trend to the solubility observed at various concentrations of trehalose (Figure 1a). The control (0T‐LP) protein digestibility was around 75%, comparable to the results reported by Alrosan et al. (2021), that is, 76%. The digestibility of 5T‐LP conjugates trehalose boosted significantly (*p *< 0.05) to ∼81.8%. It was reported by Han et al. (2023) and Alrosan et al. (2024) that trehalose was observed to increase the digestibility of proteins. Their observations suggested the potential formation of additional non‐covalent interactions during the trehalose conjugation of plant‐based protein when the trehalose concentration exceeded 2%. It was hypothesized that the combination of LPs with trehalose at higher levels is mainly caused by electrostatic interaction, hydrophobicity, and hydrogen bonds.

Moreover, Han et al. (2023) demonstrated that trehalose can affect the intermolecular forces inside the protein structure, particularly hydrogen bonds. These changes can reflect on the tertiary structure and amid I band, such as the α‐helix and the random coil. Hence, it has been suggested that trehalose can modify non‐covalent bonding, specifically hydrogen bonds, which change the stability and structure of proteins. Additionally, the disruption of these intermolecular forces could potentially influence protein folding and overall functionality. It was also reported that trehalose could increase the functionality of casein conjugates of trehalose, which can also modify the interactions between molecules that are present α‐ and β‐casein proteins (Bhat et al., 2023).

The hydroxyl groups of trehalose can displace water molecules and establish robust hydrogen bonds with proteins and other substances (Alrosan et al., 2024). These hydroxyl groups can form hydrogen bonds with the polar side chains of amino acids in proteins. By creating these bonds, trehalose can effectively replace water molecules, usually hydrogen‐bonded to the protein, thus stabilizing the protein structure. Moreover, by shielding hydrophobic regions exposed during denaturation (alkaline environments), trehalose prevents these regions from sticking together and forming aggregates (Alrosan et al., 2024). The extremely high hydrophobicity of β‐sheet structures in legume proteins can create dense, rigid, and sealed structures. These tiny structures can limit the ability of digestive enzymes to perform their duties, resulting in a decrease in the digestibility of proteins. The increased digestibility shown in Figure 1a can be attributed to the increase in β‐sheet structure, which leads to reduced protein connections and the development of smaller aggregates. Smaller aggregates enhance the accessibility of proteases to their target sites, resulting in improved digestion. Therefore, the interaction of LPs with trehalose leads to an increase in the β‐sheet content and a decrease in β‐turn content, which impacts the protein's structural properties. The structural changes induced by trehalose can improve the functional properties of the legume proteins, such as solubility and digestibility. These changes benefit various applications, including food processing and nutritional enhancement.

### 3.2 Protein solubility

Protein solubility is the ability of a protein to dissolve in water, and several variables significantly influence protein solubility, such as pH (Alrosan et al., 2022b), temperature (Jarpa‐Parra et al., 2014), and salt concentration (Tanger et al., 2020). The solubility of LPs was ∼60.75% (Figure 1a). The solubility of 5T‐LP exhibited a significant rise (*p *< 0.05), obtaining around 66.55%. The 5T‐LP can synergistically enhance water solubility by leveraging the trehalose's solubilizing properties and modifying the protein's surface properties. Trehalose can alter the surface properties of quinoa proteins, consequently enhancing their ability to water solubility. The solubility of proteins is a crucial factor in affecting their digestibility (Alrosan et al., 2024). This finding aligns with the general understanding that trehalose can interact with proteins to enhance their solubility.

Furthermore, trehalose can interact with the protein surface through hydrogen bonding and other molecular forces (Alrosan et al., 2024). These forces can lead to structural changes in the protein (Liu et al., 2023). These modifications enhanced the functionality of the proteins, making trehalose a promising additive in various food applications in the industries. The study also highlighted that trehalose's ability to improve protein solubility could significantly affect the development of new protein‐based trehalose composite. Trehalose contains multiple hydroxyl groups that can form hydrogen bonds with proteins. These bonds can modify the proteins in solution and reduce aggregation. In addition, the conjugates of trehalose can lead to a more flexible protein structure, enhancing its ability to remain in solution. The significant increase in the solubility of egg yolk proteins from 15% to 56% after trehalose conjugation, as reported by Wang et al. (2023), demonstrates the powerful effect of trehalose on protein solubility. This effect is mainly due to trehalose's ability to form hydrogen bonds, reduce protein aggregation, and stabilize protein structures, improving solubility and digestibility in various applications. Consequently, egg yolk protein's water solubility significantly improved, increasing from 14% to 62%. These modifications were hypothesized to be attributed to the ability of trehalose to form non‐covalent forces with the protein molecules, increasing the solubility of egg yolk protein.

### 3.3 Trehalose‐LP structure properties

#### 3.3.1 Secondary protein

The secondary protein structure of LPs can be identified by the arrangement of its amino acids into distinct structural structures, mainly α‐helix and β‐sheet, as well as random coil and β‐turn. Table 1 shows the results of this study indicate that trehalose has a statistically significant (*p *< 0.05) effect on the alterations in content of α‐helix and β‐sheet, random coil, and β‐turn. The protein 0T‐LP had the lowest proportion of α‐helix and random coil structures while the highest proportion of β‐sheet and β‐turn structures (Table 1). These results align with the results found by Alrosan et al. (2021). The alteration at the secondary structural level was confirmed due to conjugation with trehalose. The 5T‐LP structure transformed to reach the highest degree of β‐sheet and random coil conformation.

**TABLE 1:** Thermal stability and changes in the percentage of secondary protein components of trehalose‐conjugated lentil proteins (LPs).

Secondary protein components	Trehalose conjugation				
	LP‐T0	LP‐T1	LP‐T2	LP‐T3	LP‐T5
**β‐Sheet (∑)**	27.99 ± 0.21^e^	28.82 ± 0.37^d^	30.69 ± 0.29^c^	33.50 ± 0.12^b^	35.36 ± 0.27^a^
**RC (∑)**	21.52 ± 0.45^d^	22.48 ± 0.48^c^	24.58 ± 0.44^c^	26.67 ± 0.17^a^	27.18 ± 0.16^a^
**α‐Helix (∑)**	21.80 ± 0.17^a^	20.70 ± 0.40^b^	19.59 ± 0.31^c^	18.95 ± 0.11^d^	17.20 ± 0.25^e^
**β‐Turn (∑)**	28.68 ± 0.84^a^	27.99 ± 0.29^a^	25.13 ± 0.22^b^	20.87 ± 0.20^c^	20.24 ± 0.44^c^
**T_s_**	107.2^e^	108.6^d^	109.3^c^	10.9.8^b^	110.3^a^

*Note*: Means (*n* = 3) with different superscripts in the same row differ significantly (*p *< 0.05). Control (0T‐LP) represents the absence of trehalose conjugation with the LPs. Meanwhile, 1T‐LP, 2T‐LP, 3T‐LP, and 5T‐LP represent LP conjugated with trehalose at 1%, 2%, 3%, and 5% (w/w), respectively.

Abbreviation: T_s_, thermal stability.

In contrast, the α‐helix and β‐turn conformations were found to reside at the lowest level. The α‐helix content of 0T‐LP decreased significantly (*p *< 0.05) from 21.80% ± 0.17% to 17.20% ± 0.25% at 5% of trehalose. It was reported by Cui et al. (2021) that adding trehalose can modify the component of the secondary protein structure of pea proteins by altering the percentage of α‐helix and β‐sheet. This modification resulted in pea protein isolates conjugated with trehalose to increase water solubility. The presence of β‐sheet structures in proteins can influence their water solubility due to the hydrophobic structure of the protein's interior. The β‐sheet content of LPs exhibited a statistically significant increase (*p *< 0.05) from 27.99% to 35.36% after treated with 5% (w/w) trehalose.

The β‐turn percentage decreased significantly (*p *< 0.05) from 28.68% to 20.24% at 5% (w/w) of trehalose. The significant drop in the percentage of β‐turns provides compelling evidence of the conformational changes in LPs, particularly water solubility. Previous investigations have shown that molecular factors, such as hydrogen bonds and electrostatic and hydrophobic interactions, can change the structure of proteins following conjugation with disaccharides (Alrosan et al., 2024; Cui et al., 2021). Moreover, the random coil of LPs exhibited a significant increase (*p *< 0.05) from 21.52 ± 0.45 to 27.18 ± 0.16 following treatment with a 5% (w/w) concentration of trehalose. According to Alrosan et al. (2024), it has been observed that trehalose has an impact on increasing the random coil structure of plant protein following treatment. These findings suggest that alterations in the proportion of secondary protein structure may influence protein digestibility, resulting in improved protein digestibility. It was reported by Liu et al. (2023) and Alrosan et al. (2024) that changes in the secondary protein structure can lead to increased water solubility and protein digestibility.

**FIGURE 2:**
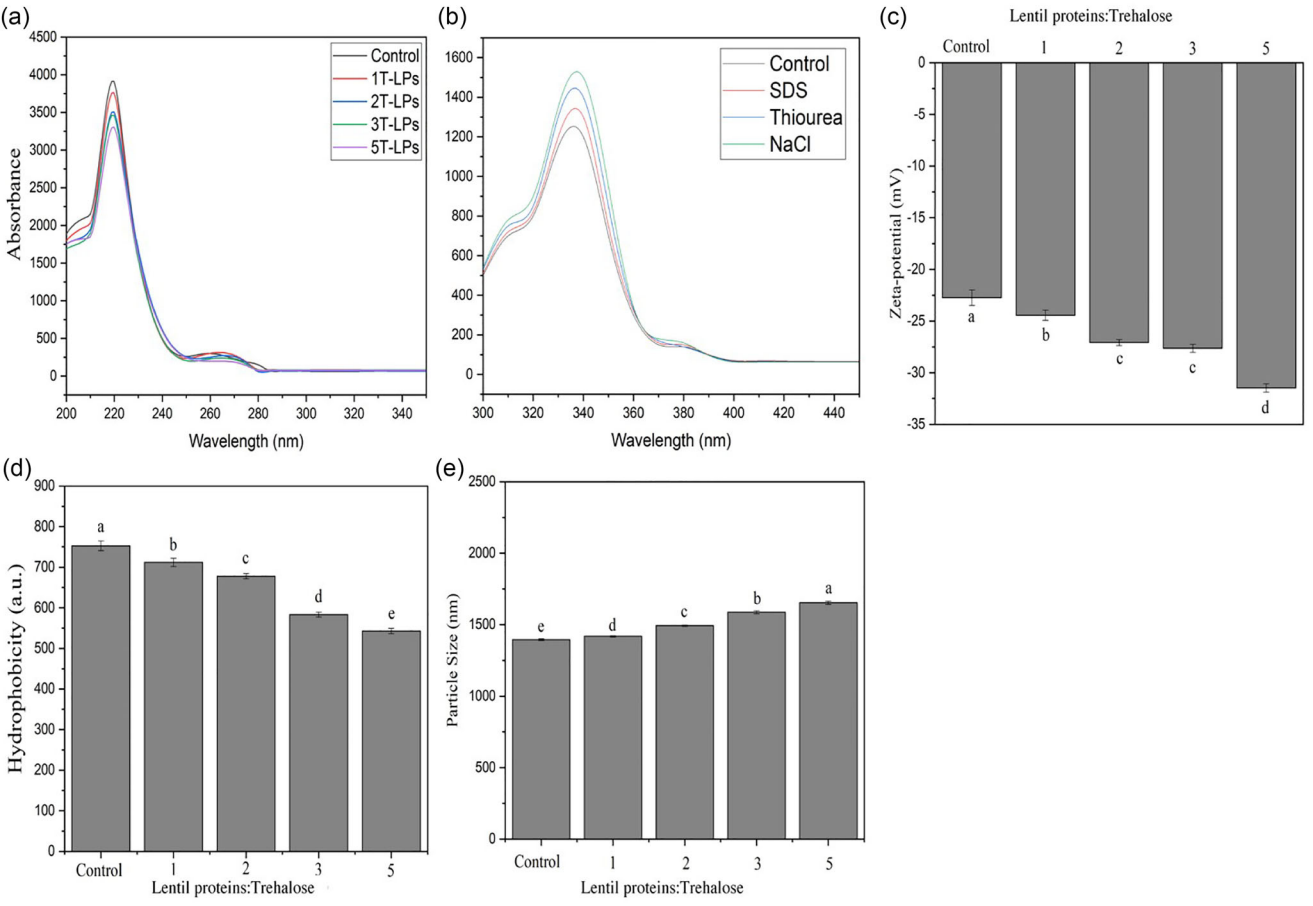
The trehalose‐conjugated lentil proteins (LPs) were evaluated for alterations in the (a) UV absorbance, (b) fluorescence intensity (molecular forces), (c) zeta potential, (d) surface hydrophobicity, and (e) particle size of trehalose‐conjugated LPs. LPs that have been conjugated with trehalose at 1, 2, 3, and 5% (w/w) are represented by the 1T‐LP, 2T‐LP, 3T‐LP, and 5T‐LP, respectively, whereas the control (0T‐LP) indicates that no trehalose conjugation with LPs is present.

Figure 1c shows the changes in the amide I and II bands within the infrared spectrum during the interaction between LPs and trehalose. These modifications indicate changes in the conformation structure of the protein. Additionally, shifts in the intensity of specific peaks can reveal the formation of new chemical bonds or changes in hydrogen bonding patterns between the protein and sugar molecules. The peak around 1535.3 cm^−^¹ is attributed to the amide II band, which corresponds to N–H bending and C–N stretching vibrations, and the maximum intensity occurs at approximately 1236 cm^−1^, which is between the N–H bending and C–N stretching bands. Changes in these peaks indicate an interaction between the sugars and protein that affects the shift in these peaks (Figure 2a). This provides strong evidence for the conjugation between trehalose and LPs.

The peaks at 3300 cm^−^¹ are related to the variation stretching of the OH group (Jiang et al., 2022). As shown in Figure 2a, shifting in the peak 3300 cm^−^¹ was observed after the conjugated LPs and trehalose. On the other hand, the absorption peak at 1031.9 cm^−^¹ in T‐LP is attributed to specific molecular vibrations, including those associated with C–O stretching, C–H bending, and C–C bonds. These indicated that trehalose is present in the T‐LP and its effect on the structure of conjugates (Figure 2a). The peak at 3300 cm^−^¹ (LPs) and 3388 cm^−1^ (T‐LP) were shifted with the increase in the trehalose concentration. The shift toward higher wavenumbers (from 3300.4 to 3308 cm^−^¹) can indicate an increase in hydrogen bonding strength or changes in the electronic environment surrounding the functional groups responsible for these peaks. Moreover, the stretching vibrations of C–O bonds typically occur in the region around 1300 cm^−^¹ (Figure 1c). These vibrations involve the stretching motion of the bond between a carbon atom and an oxygen atom, which is found in functional groups such as carbonyl groups (C = O) in organic molecules.

#### 3.3.2 Tertiary protein structure

Tryptophan fluorescence (at 280 nm) arises from the intrinsic fluorescence of the indole ring present in the tryptophan side chain. The tryptophan residues can emit light in the visible range, with a maximum emission wavelength of around 330–340 nm (Figure 1d). The alterations in fluorescence intensity observed at different trehalose concentrations (up to 5%, w/w) suggest changes in the local environment surrounding the tryptophan residues in LPs. Tryptophan residues (intrinsic fluorophores) change their fluorescence properties in response to alterations in their microenvironment, such as solvent polarity, exposure to water, and interactions with nearby residues based on the trehalose–LP interaction degree. The fluorescence intensity of tryptophan decreased as the trehalose content increased in the LPs. The outcomes agree with the findings by Wang et al. (2019), whereby the presence of external compounds in proteins has a substantial effect on their tertiary structure. However, a decrease in fluorescence intensity does not necessarily imply a reduction in the percentage of tryptophan residues within the protein. This decrease could be due to the changes in the fluorescence properties of the tryptophan residues in LPs or alterations in their microenvironment. Several investigations conducted by Alrosan et al. (2024) and Han et al. (2023) have shown that trehalose plays a crucial role in modifying the structure of proteins during conjugation and might enhance water solubility (Alrosan et al., 2024; Cui et al., 2021; Han et al., 2023; Liu et al., 2023) and protein digestibility (Alrosan et al., 2024; Liu et al., 2023). The fluorescence intensity of LP‐trehalose reduced (Figure 1c), indicating that certain portions of the tryptophan residue in the protein macro‐molecules were exposed to the polar environment (Nian et al., 2019) and the formation of a more open protein structure. Recent studies reported that modifications in the tertiary structure of protein can let to enhance water solubility (Alrosan et al., 2024; Han et al., 2023; Jiang et al., 2022; Liu et al., 2023; Wang et al., 2023) and protein digestibility (Alrosan et al., 2024; Liu et al., 2023; Qu et al., 2018). During lyophilization, the removal of water can lead to the destabilization of the protein structure, causing it to unfold (Alrosan et al., 2022b) and expose hydrophobic groups and amino acids, including tryptophan, that are normally buried inside the protein's core. The exposure of hydrophobic groups can promote interactions between protein molecules, leading to aggregation. Hydrophobic interactions drive these proteins to form aggregates to minimize their exposure to the aqueous environment (Alrosan et al., 2022b).

#### 3.3.3 Protein conformation

The conformation of the protein significantly influences the functional characteristics of proteins. It can be influenced by various factors, including temperature, pH, and solvent conditions (Alrosan et al., 2022b; Wang et al., 2023). Changes in protein conformation can affect its stability, activity, and interactions with other molecules. The UV spectra in the wavelength range of 190–350 nm, narrowing in on the aromatic side chains of phenylalanine, tyrosine, and tryptophan (Wang et al., 2019). These amino acids can be observed within this spectrum (Han et al., 2023). Figure 1d shows a spectrum whereby the amount has decreased with the increase in trehalose concentration in the conjugates.

Trehalose acts as a chemical chaperone that assists proteins undergo structural modifications, consequently improving their functionality, particularly in water solubility (Alrosan et al., 2024; Han et al., 2023; Jiang et al., 2022; Liu et al., 2023; Wang et al., 2023) and protein digestibility (Alrosan et al., 2024; Liu et al., 2023; Qu et al., 2018). The concentration‐dependent nature of these effects suggests that trehalose likely exerts its influence through specific interactions with proteins, which become more pronounced at higher concentrations (Wang et al., 2023). Recent studies reported by Bhat et al. (2023) and Alrosan et al. (2024) that changes in protein conformations can alter protein functionalities. The structural changes in trehalose‐LP conjugates can cause the entire structured arrangement of LPs to unfold, resulting in greater exposure to aromatic hydrophobic amino acids. Intrinsic tryptophan can validate this impact on the tertiary structure of LPs. The interaction between trehalose and LP can disrupt the tightly packed protein structure (Bhat et al., 2023) leading to unfolding. This disruption allows for the exposure of buried hydrophobic regions and aromatic amino acids, such as tryptophan. Hydrophobic amino acids, previously shielded within the protein's core, become more exposed due to unfolding. This exposure can be detected by changes in the protein's structure properties (Sun et al., 2023).

On the other hand, several investigations have shown that the solubility of plant proteins increased after modifying the protein's conformation and secondary structure (Alrosan et al., 2024; Jiang et al., 2022). A recent study by Alrosan et al. (2022b) suggested that pH‐shifting treatments can facilitate the unfolding and subsequent re‐engagement of protein structures through non‐covalent forces and could enhance the functional properties of the protein (Wang et al., 2019). The re‐engagement of protein structures is primarily driven by non‐covalent forces such as hydrogen bonds, ionic interactions, van der Waals forces, and hydrophobic interactions. The unfolding and subsequent refolding can reduce protein aggregation but enhance water solubility since the process can break up large aggregates and promote a more dispersed state.

#### 3.3.4 Non‐covalent interactions

Non‐covalent interactions are crucial in forming conjugates, complexes formed from two or more molecules, including proteins and disaccharides (Alrosan et al., 2024; Cui et al., 2021). The previous research suggested that changes in the intensity of hydrophobic and electrostatic interactions, as well as hydrogen bonds, could have caused these structural modifications (Alrosan et al., 2024; Cui et al., 2021). According to the results (Figure 2b), electrostatic interactions significantly form conjugates between trehalose and LPs, surpassing hydrogen bonds and hydrophobic interactions. It was reported by Alrosan et al. (2024) and Cui et al. (2021) that non‐covalent bonding, including electrostatic interactions, hydrogen bonds, and hydrophobic interactions, has a vital role in the formation of complex composites between disaccharides and plant proteins. Moreover, Cui et al. (2021) showed that hydrogen bonds may generate conjugates between pea protein isolates and disaccharides (trehalose) and increase their solubility. Our results emphasize the potential enhancement of functionality in complex composites by forming electrostatic interactions, hydrogen bonds, and hydrophobic interactions between egg yolk and disaccharides. In addition, our findings also suggest that different non‐covalent bonding mechanisms can be crucial in creating conjugates with improved properties.

### 3.4 Surface properties

#### 3.4.1 Surface charge

Surface charge can also influence hydrogen bonding capabilities. Charged residues can enhance the ability of nearby polar groups to participate in hydrogen bonding by creating a favorable electrostatic environment, which results in improved functional properties of proteins (Alrosan et al., 2022b; Cui et al., 2021). In this study, 0T‐LP has a surface charge of approximately −22.40 mV (Figure 2b). This finding is consistent with the results obtained by Alrosan et al. (2024). The surface charge of 5T‐LP increased significantly (*p *< 0.05) to approximately −31.93 mV. The decrease in the surface charge of LPs upon conjugation with trehalose indicates that electrostatic interactions play a significant role. This change suggests that the binding of trehalose to LPs results in an overall increase in the negative charge of the conjugates. A study by Jiang et al. (2022) showed that the conjugates between inulin proteins could be due to electrostatic interactions. The interaction between trehalose and LPs may be similar to the electrostatic interactions observed in the conjugates between trehalose and LPs.

#### 3.4.2 Surface hydrophobicity

Surface hydrophobicity is an essential consideration for evaluating the interactions between proteins and disaccharides in complex composites (Alrosan et al., 2024; Alrosan et al., 2022b; Cui et al., 2021; Jiang et al., 2022). The surface hydrophobicity of 0T‐LP is roughly 678, indicating that the water solubility of LP is poor in an aqueous solution. Excessive surface hydrophobicity often leads to protein aggregation and precipitation, reducing solubility (Liu et al., 2023). These results align with those reported by Alrosan et al. (2024) and Jarpa‐Parra et al. (2017) in their studies. The conjugation of trehalose with LPs decreased the surface hydrophobicity because trehalose acted as a stabilizer by forming hydrogen bonds with the surface residues of proteins (Bhat et al., 2023). This interaction can shield hydrophobic residues from the surrounding aqueous environment, reducing the overall surface hydrophobicity of the protein. In general, trehalose might alter the conformation of the proteins and prevent aggregation or denaturation by decreasing the surface hydrophobicity. The 5T‐LP has surface hydrophobicity around ∼543 at a ratio of 5% (w/w). Reducing surface hydrophobicity can improve solubility and prevent aggregation, enhancing the protein's functionality (Bhat et al., 2023; Liu et al., 2023).

### 3.5 Particle size

The LPs have a smaller (*p *< 0.05) particle size compared to the T‐LP (Figure 2e). This larger particle size can lead to improved functionality of LP‐conjugated trehalose, as well as, it may also enhance the overall bioavailability of the protein for better absorption. The particle size of the T‐LP exhibited a significant rise (*p *< 0.05) with the increasing trehalose concentration. At the ratio of 5 % of the trehalose, the T‐LP has the largest potential size. It is possible to attribute this to a rise in the viscosity of the solution, which, in turn, led to the formation of a soluble complex composite (Jiang et al., 2022). The previous research has suggested that the interaction between proteins with either polysaccharides (Han et al., 2023) or disaccharides (Alrosan et al., 2024; Cui et al., 2021) may raise the particle size of complex composite. This particle size increase can influence the conjugates’ overall functional properties, potentially affecting their applications across various industries. Understanding these interactions is crucial for optimizing the formulation and performance of polysaccharide, disaccharide, and protein‐based products.

### 3.6 Thermal stability

The thermal stability of T‐LP at range (107.2–110.3°C) and the thermal stability of 0T‐LP are at their lowest points at around 107.2°C. This finding aligns with results reported by Parolia et al. (2022) that the thermal stability of LPs is around 105.9°C (Table 1). The thermal stability of T‐LPs has been improved with increasing ratios of trehalose in the conjugates. 5T‐LP has the highest value of thermal stability, around 110.3°C. This research indicates that trehalose has a significant effect in increasing the resistance of LPs to temperature. Recent studies have found that the conjugation of disaccharides (Cui et al., 2021) and polysaccharides (Han et al., 2023) with proteins can significantly influence the resistance to the heat of the proteins. Hence, the interactions between trehalose and LPs have effectively formed conjugates between LPs and trehalose, enhancing the thermal stability of the conjugates.

## 4 CONCLUSION

In this study, we have investigated the feasibility of incorporating an agent (trehalose) during the pH‐shifting technique in an alkaline environment (12.0) and recycling the pH to a neutral environment (pH 7.0). We have also evaluated the multi‐structure and surface properties and molecular forces that govern protein‐disaccharide interactions of the LP‐conjugated trehalose. Conjugation with trehalose alters the properties of protein structure, including conformation, secondary structure, and tertiary structure. Increasing the trehalose concentration in the conjugates also changed the surface charge and hydrophobicity. The soluble protein‐trehalose composite conjugates have improved water solubility and digestibility. Furthermore, the conjugation with trehalose enhanced the thermal stability of the protein structure, making it more resistant to denaturation. Overall, incorporating trehalose into protein conjugates shows promise for improving their functionality and potential applications in various industries. Moreover, the results of this study indicate that pH‐shifting might be an encouraging method for enhancing the functionality of plant proteins. The improved solubility and digestibility of the conjugates could have potential applications in the food industry for developing new functional food products.

## AUTHOR CONTRIBUTIONS

**Mohammad Alrosan**: Conceptualization; methodology; software; formal analysis; supervision; project administration; writing—review and editing; writing—original draft; data curation; resources; visualization. **Sofyan Maghaydah**: Writing—review and editing; software; visualization. **Ali Al‐Qaisi**: Methodology; writing—original draft; investigation. **Ali Madi Almajwal**: Writing—review and editing; writing—original draft; project administration; formal analysis; funding acquisition. **Muhammad H. Alu'datt**: Writing—review and editing. **Farah R. Al Qudsi**: Validation; investigation. **Thuan‐Chew Tan**: Supervision; writing—review and editing; writing—original draft. **Ammar A. Razzak Mahmood**: Writing—review and editing; writing—original draft; formal analysis.

## CONFLICT OF INTEREST STATEMENT

The authors reported no conflicts of interest.
